# The Association Between FT3 With the Outcome and Inflammation/Coagulopathy/Fibrinolysis of COVID-19

**DOI:** 10.3389/fendo.2022.877010

**Published:** 2022-06-03

**Authors:** Jiayi Deng, Siye Zhang, Fei Peng, Quan Zhang, Yi Li, Yanjun Zhong

**Affiliations:** ^1^ Critical Care Medicine, The Second Xiangya Hospital, Central South University, Changsha, China; ^2^ Critical Care Medicine, Hunan Provincial People’s Hospital, Changsha, China; ^3^ Department of Respiratory, The Second Xiangya Hospital, Central South University, Changsha, China; ^4^ Department of Cardiology, The Second Xiangya Hospital, Central South University, Changsha, China

**Keywords:** COVID-19, FT3, euthyroid sick syndrome, outcome, inflammation/coagulopathy/fibrinolysis

## Abstract

**Background:**

The coronavirus disease 2019 (COVID-19) pandemic has caused substantial threats to people’s physical health and lives, claiming the lives of over 5 million people worldwide. It is imperative to identify the disease severity and intervene with effective therapy as early as possible. Previous studies have shown that low free triiodothyronine (FT3) may possess the predictive value on COVID-19 prognosis.

**Methods:**

In this retrospective cohort study, 15-day clinical and laboratory data of 186 hospitalized patients of COVID-19 after admission were analyzed. Groups were based on the disease severity of COVID-19, survival or non-survival, and presence or absence of euthyroid sick syndrome (ESS). Categorical variables were compared with the chi-square test or Fisher’s exact test. Continuous variables were tested by Wilcoxon rank-sum test for the non-normal distribution. Spearman correlations were used to assess the correlations between FT3 with clinic parameters of multiple time points.

**Results:**

The non-survival patients had significant lower levels of FT3 (3.24 ± 0.42 vs. 4.19 ± 0.08 pmol/L, *p* < 0.05) and thyroid-stimulating hormone (TSH) (0.69 ± 0.19 vs. 2.32 ± 0.2 uIU/ml, *p* < 0.05), and the FT3 of severe patients was significantly lower than that of non-severe patients (3.67 ± 0.14 vs. 4.33 ± 0.09 pmol/L, *p* < 0.05). Fifty-nine cases of COVID-19 patients were diagnosed with ESS. Compared with non-ESS patients, those with ESS were older and had higher proportions of fever, shortness of breath, hypertension, diabetes, severe disease, and mortality. In addition, the correlation analysis between FT3 and clinical parameters showed that FT3 were positively related to the lymphocyte count and albumin and negatively correlated with C-reactive protein, erythrocyte sedimentation rate, and D-dimer at all time points in the first 15 days after admission.

**Conclusion:**

Low FT3 had a significant predictive value on the prognosis of COVID-19 patients, and FT3 was significantly related with clinic parameters of inflammation/coagulopathy/fibrinolysis.

## Introduction

Coronavirus disease 2019 (COVID-19) caused by the etiological agent of severe acute respiratory syndrome coronavirus 2 (SARS-CoV-2) (1) has swept the world in recent years. From 2019 to the present, COVID-19 remains prevalent. As of April 1, 2022, there were 486,761,597 confirmed cases and 6,142,735 deaths reported (2). The issue of prognosis in COVID-19 patients have received considerable critical attention.

COVID-19 can damage many organs throughout the body, and there has been a substantial number of deaths caused by COVID-19. It is necessary to find biomarkers of disease severity and death to identify earlier, intervene effectively, and save lives ultimately. Previous studies suggested that changes in thyroid function relevant parameters have been thought to be significant in COVID-19 patients (3–5). However, the effects of COVID-19 on the thyroid axis and the crosstalk between the thyroid axis and inflammation/coagulopathy/fibrinolysis during the SARS-CoV-2 infection remain not very clear. To our knowledge, COVID-19 infection can cause cytokine storm, impair the immunology system, and even result in diffuse intravascular coagulation (DIC), while the thyroid gland is a neuroendocrine organ participating in immune regulation (6, 7).

The thyroid gland could be a target of SARS-CoV-2, leading to possible detrimental consequences on thyroid function. In addition, the hypothalamus–pituitary–thyroid axis could be affected (8). The pathogenesis of euthyroid sick syndrome (ESS) have been discussed in some studies (9–11), and cytokines released during illness are considered a major determinant of ESS, since they affect a variety of genes involved in the metabolism of thyroid hormones (9). Meanwhile, ESS may be the result of a stress response or a direct viral attack on the gland. The latter was supported by the mRNA encoding for the angiotensin-converting enzyme 2 (ACE 2) receptor expressed in follicular cells, making them a potential target for SARS-COV-2 entry (12). However, the determination of the causal relationship of thyroid diseases and virus infection, if any, still requires extensive clinical research. Particularly, previous studies hint that low serum levels of free T3 (FT3) might be of a practical predictive value in prognosis (3, 13–15). The primary purpose of our research was to further clarify the predictive value of FT3 in COVID-19 outcome and the potential mechanisms with regard to the dynamic process of inflammation/coagulopathy/fibrinolysis during COVID-19 infection.

We conducted a retrospective study in COVID-19 patients; the demographic and clinical characteristics and laboratory findings of 186 patients included were assessed. We found that the severe and non-survival patients with COVID-19 had a lower serum FT3. Meanwhile, the patients who were older and had hypertension or diabetes were more apt to have a lower serum FT3. Besides, we concluded that FT3 was significantly related with inflammation/coagulopathy/fibrinolysis.

## Methods

### Design and Participants

In this retrospective cohort study, we enrolled 186 confirmed cases of COVID-19, who were hospitalized successively in the Public Health Treatment Center of Changsha and Zhongfa Hospital, Wuhan, China, as of March 26, 2020. The patients who had a history of pituitary dysfunction and/or thyroid dysfunction were excluded. According to the endpoints of patients during the study period, the enrolled patients were stratified into a survival group and a non-survival group and a severe group and a non-severe group. The patients with the following conditions were classified as the severe group: (1) respiratory rate ≥ 30/min; (2) oxygen saturation ≤ 93%; (3) PaO_2_ (partial pressure of arterial oxygen)/FiO_2_ (fraction of inspired oxygen) ≤ 300 mmHg; (4) progression of lung lesions exceeding 50% within 24–48 h; (5) mechanical ventilation was implemented; (6) shock; and (7) intensive care unit admission. Based on the levels of serum FT3, patients were divided into ESS group [FT3 <2.3 pg/ml **+** normal or low thyroid-stimulating hormone (TSH)] and non-ESS group (3). According to the nearby days without statistically significant differences in laboratory findings, we divided them into five different periods, with 3 days for each phase: T0 (D0–D2), T1 (D3–D5), T2 (D6–D8), T3 (D9–D11), and T4 (D12–D14). This study was subjected to the approval by the institutional ethics board of the Second Xiangya Hospital of Central South University (No. 2020001).

### Data Collection

For this study, we analyzed the data collected from the electronic medical record. The following data were reviewed and extracted: demographic data and chronic comorbidities at the beginning of admission, clinical symptoms, outcomes, and relevant laboratory parameters at different time points, including thyroid function, routine blood examinations, coagulation, liver function, renal function, and inflammatory parameters. Thyroid function indexes, including FT3, free thyroxine (FT4), and TSH levels were measured using chemiluminescence immunoassay. All patients had blood sampling done at admission to determine TSH (normal range, 0.27–4.2 mIU/ml), FT4 (normal range, 12–22 pmol/L), and FT3 (normal range, 3.5–6.5 pmol/L) levels.

### Statistical Analysis

In this study, all continuous variables were described using the mean ± standard error and analyzed using t-test if the distribution is normal, or otherwise using Wilcoxon rank-sum test. Categorical variables were expressed as frequencies and analyzed using Fisher’s exact test and chi-square test. Spearman correlations were performed to assess the correlations between continuous variables. A *p*-value of <0.05 was considered statistically significant. All analyses were conducted by SPSS 26.0 or R language.

## Results

In this study, 186 confirmed cases of COVID-19 were included. Among them, 14 patients died, and 61 patients were severe cases. We compared the thyroid-function-related indicators (FT3, FT4, and TSH) in different groups according to the survival and disease severity of the COVID-19 patients. The patients of the non-survival group had lower levels of FT3 and TSH than survival patients (3.24 ± 0.42 vs. 4.19 ± 0.08, *p* < 0.05 and 0.69 ± 0.19 vs. 2.32 ± 0.2, *p* < 0.05). Similarly, the patients of the severe group had lower FT3 than non-severe patients (3.67 ± 0.14 vs. 4.33 ± 0.09, *p* < 0.05). Nonetheless, there was no significant difference in the terms of FT4 (**Table 1**).

**Table 1:** Thyroid function in different groups according to survival and disease severity.

	Survival (n = 172)	Non-survival (n = 14)	Non-severe (n = 125)	Severe (n = 61)
FT3 (pmol/L)	4.19 ± 0.08	3.24 ± 0.42*	4.33 ± 0.09	3.67 ± 0.14*
FT4 (pmol/L)	16.17 ± 0.35	16.39 ± 1.34	16.04 ± 0.32	16.49 ± 0.8
TSH (uIU/ml)	2.32 ± 0.2	0.69 ± 0.19*	2.06 ± 0.14	2.48 ± 0.51

*Significant difference vs. control.

Among 186 patients, 59 cases were diagnosed as ESS. Cough [28 (86.4%)], fever [49 (83.1%)], and fatigue [25 (42.4%)] were the common symptoms in patients with ESS. Patients of ESS group were significantly older than non-ESS patients (58.64 ± 1.70 vs. 46.94 ± 1.41, *p* < 0.001). Furthermore, patients of the ESS group were more likely to have symptoms of shortness of breath and fever than non-ESS patients (47.5% vs. 26.0%, *p* = 0.004 and 83.1% vs. 64.6%, *p* = 0.010). ESS patients have higher proportions of hypertension and diabetes, compared with the non-ESS patients (28.8% vs. 10.2%, *p* = 0.001 and 22.0% vs. 4.7%, *p* < 0.001). Meanwhile, the clinical outcomes were significantly worse in the ESS group with higher occurrences of severe disease and mortality (55.9% vs. 22.0%, *p* < 0.001 and 18.6% vs. 2.4%, *p* < 0.001) (**Table 2**). It revealed the dynamic process of the disease and the differences between ESS and non-ESS groups with respect to relevant laboratory parameters. There was a significant trend of increasing inflammatory biomarkers in the ESS patients. White blood cell (WBC) count showed an upward trend from T0 to T4 (except for T2); lymphocytes (Lys) at T1 was lowest, and procalcitonin was increasing from T0 to T4 (except for T1 and T4, respectively); erythrocyte sedimentation rate (ESR) had two peaks at T1 and T3; and C-reactive protein (CRP) had a decreasing tendency, while T4 was significantly elevated. About coagulation indicators, D-dimer and activated partial thromboplastin time (APTT) increased from T0 to T4 (except for T2), and platelet count increased from T0 to T3, with prothrombin time (PT) and fibrin degradation product (FDP) decreasing from T0 to T4 (except for T4 and T3, respectively) (**Table 3**).

**Table 2:** Baseline characteristics of patients with ESS and non-ESS.

	ESS group (n = 59)	Non-ESS group (n = 127)	All patients (n = 186)	*p* value
Gender (male/female)	25/34	65/62	90 (48.4%)	0.263
Age, y, M (IQR)	58.64 ± 1.70	46.94 ± 1.41	50.65 ± 1.17	<0.001*
**Symptoms**				
Fever (n, %)	49 (83.1%)	82 (64.6%)	131 (70.4%)	0.010*
Fatigue (n, %)	25 (42.4%)	44 (34.6%)	69 (37.1%)	0.310
Cough (n, %)	51 (86.4%)	102 (80.3%)	153 (82.3%)	0.309
Shortness of breath (n, %)	28 (47.5%)	33 (26.0%)	61 (32.8%)	0.004*
Diarrhea (n, %)	13 (22.0%)	27 (21.3%)	40 (21.5%)	0.905
Abdominal pain (n, %)	4 (6.8%)	5 (3.9%)	9 (4.8%)	0.636
Myalgia (n, %)	5 (8.5%)	18 (14.2%)	23 (12.4%)	0.272
Headache (n, %)	13 (22.0%)	27 (21.3%)	26 (14.0%)	0.911
**Comorbidities**				
Hypertension (n, %)	17 (28.8%)	13 (10.2%)	30 (16.1%)	0.001*
Cardiovascular (n, %)	6 (10.2%)	6 (4.7%)	12 (6.5%)	0.277
Diabetes (n, %)	13 (22.0%)	6 (4.7%)	19 (10.2%)	<0.001*
**Outcomes**				
Severe disease (n, %)	33 (55.9%)	28 (22.0%)	61 (32.8%)	<0.001*
Mortality (n, %)	11 (18.6%)	3 (2.4%)	14 (7.5%)	<0.001*

*means P < 0.05.

**Table 3:** Laboratory findings of patients with ESS and non-ESS at different time points after admission.

	T0 (D0–D2)		T1 (D3–D5)		T2 (D6–D8)		T3 (D9–D11)		T4 (D12–D14)	
	Non-ESS	ESS	Non-ESS	ESS	Non-ESS	ESS	Non-ESS	ESS	Non-ESS	ESS
WBC	5.22 ± 3.03	7.37 ± 4.86*	6.01 ± 2.78	8.12 ± 5.69*	6.57 ± 3.04	7.97 ± 4.63*	7.26 ± 4.24	8.97 ± 4.85	7.39 ± 2.88	10.52 ± 5.95*
HGB	129.46 ± 16.17	124.41 ± 16.17	130.04 ± 16.8	123.04 ± 15.97	127.45 ± 17.80	120.94 ± 17.29	123.91 ± 18.99	118.41 ± 16.45	123.5 ± 20.2	115.88 ± 17.59
PLT	197.63 ± 71.74	177.26 ± 86.43	216.97 ± 78.27	193.09 ± 112.23*	236.88 ± 86.10	207.21 ± 97.26	244.62 ± 88.66	242.18 ± 100.98	254.05 ± 99.48	236.33 ± 135.04
Lys	1.39 ± 0.6	0.84 ± 0.36*	1.4 ± 0.63	0.78 ± 0.41*	1.45 ± 0.58	0.85 ± 0.42*	1.55 ± 0.64	1.14 ± 0.55	1.53 ± 0.61	1.26 ± 0.56
CRP	20.16 ± 30.51	58.96 ± 54.88*	23.65 ± 37.5	49.18 ± 46.99*	18.42 ± 29.26	40.64 ± 60.27*	7.08 ± 12.8	29.84 ± 41.81*	9.28 ± 20.15	55.45 ± 56.86*
PCT	0.2 ± 1.14	0.2 ± 0.39	0.15 ± 0.78	0.28 ± 0.97	0.61 ± 4	1.12 ± 6.21	0.05 ± 0.31	1.67 ± 6.73*	0.37 ± 1.63	1.29 ± 3.71*
ESR	38.84 ± 29.39	54.43 ± 31.46	44.09 ± 31.35	71.94 ± 27.07	57.73 ± 34.68	69.82 ± 33.24	55.04 ± 35.66	80.58 ± 19.9*	54.93 ± 33.34	65.34 ± 39.34
PT	11.99 ± 1.39	13.95 ± 4.66*	12.92 ± 9.39	13.52 ± 4.82	11.39 ± 1.81	13.05 ± 5.61*	11.36 ± 2.11	12.28 ± 3.21*	11.4 ± 2.11	13.41 ± 2.86*
APTT	33.95 ± 5.05	34.81 ± 7.96*	33.84 ± 5.6	36.02 ± 9.41*	32.93 ± 4.19	31.76 ± 11.79*	33 ± 5.71	32.15 ± 6.93	32.87 ± 6.6	34.49 ± 9.33
FDP	3.62 ± 1.14	4.14 ± 1.46	3.88 ± 1.21	3.9 ± 1.54	3.59 ± 0.99	3.74 ± 1.46	3.29 ± 0.82	3.78 ± 1.33	3.03 ± 0.66	3.53 ± 1.92*
D-dimer	0.45 ± 0.57	3.58 ± 5.88*	0.69 ± 2.27	3.74 ± 5.76*	0.87 ± 3.17	2.48 ± 4.07*	0.84 ± 2.82	4.06 ± 5.88*	1.03 ± 1.78	4.81 ± 5.14*
ALT	22.6 ± 13.13	25.81 ± 19.22*	22.06 ± 16.51	30.34 ± 46.26*	37.47 ± 63.59	35.02 ± 49.42	39.4 ± 43.19	49.28 ± 96.12	43.96 ± 37.21	53.24 ± 62.43*
AST	25.52 ± 10.12	33.23 ± 18.74*	24.54 ± 10.49	32.98 ± 23.79*	38.25 ± 96.25	47.51 ± 97.3	31.37 ± 30.38	38.01 ± 49.67	28.84 ± 14.89	48.52 ± 67.37*
TBil	11.42 ± 5.27	23.52 ± 58.38*	14.55 ± 8.75	24.56 ± 50.6*	10.95 ± 6.8	23.49 ± 56.38*	10.44 ± 6.43	12.56 ± 7.43	12.1 ± 9.62	18.62 ± 20.89
IBil	9.19 ± 0.27	11.15 ± 2.89	8.91 ± 0.52	11.02 ± 2.27	6.89 ± 0.37	10.80 ± 3.56	6.44 ± 0.32	7.16 ± 0.58	6.61 ± 0.42	7.93 ± 0.97
ALB	37.94 ± 4.19	33.45 ± 3.74	37.9 ± 4.49	32.98 ± 4.41	38.38 ± 5.14	33.2 ± 5.38	38.57 ± 5.86	33.26 ± 5.01	39.4 ± 5.52	33.78 ± 4.51
Cr	56.76 ± 18.23	70.66 ± 98.71*	62.87 ± 20.58	80.42 ± 116.85*	64.73 ± 63.21	62.98 ± 42.76	69.03 ± 77.75	84.56 ± 143.39	66.72 ± 52.97	103.72 ± 209.63
BUN	4.35 ± 1.62	6.22 ± 4.36*	4.75 ± 2.07	7.46 ± 5.40*	5.54 ± 4.03	7.04 ± 5.74	9.52 ± 30.71	7.13 ± 4.18	6.93 ± 6.57	7.86 ± 5.4

*Significant difference vs. control.

ESS, euthyroid sick syndrome; WBC, white blood cell; HGB, hemoglobin; PLT, platelet; Lys, lymphocytes; CRP, C-reactive protein; PCT, procalcitonin; ESR, erythrocyte sedimentation rate; PT, prothrombin time; APTT, activated partial thromboplastin time; FDP, fibrin degradation product; ALT, alanine aminotranspherase; AST, aspartate aminotransferase; TBil, total bilirubin; IBil, indirect bilirubin; ALB, albumin; Cr, serum creatinine; BUN, urea nitrogen.

A closer inspection of the data showed that the differences between ESS and non-ESS groups were highlighted. The WBC count, CRP, and D-dimer were significantly higher during the study period, whereas Lys (from T0 to T2) and APTT (T2) were obviously lower in ESS patients than those of non-ESS patients. Besides, platelet count (T1), procalcitonin (T3, T4), APTT (T1, T2), and PDF (T4) were also significantly higher in ESS patients. In terms of the liver function indicators and the levels of alanine aminotranspherase (ALT) and aspartate aminotransferase (AST) (T0, T1, T4), total bilirubin (TBil) (T1, T2, T3) was obviously higher in patients with ESS, while the indirect bilirubin had no significant differences. With respect to kidney function, patients with ESS had a higher level of serum creatinine (Cr) and urea nitrogen (BUN) at T0 and T1 (**Table 3**).

**Figure 1:**
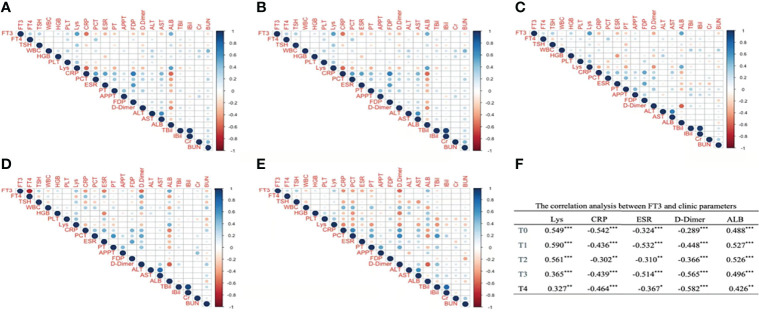
The correlation analysis between FT3 and clinic parameters at different time points after admission. Panel **(A)** corresponds to T0, Panel **(B)** corresponds to T1, Panel **(C)** corresponds to T2, Panel **(D)** corresponds to T3, Panel **(E)** corresponds to T4. **(A–E)** Digits represents Pearson correlation coefficients, filled color indicates significant correlation, blue represents positive correlation, red represents negative correlation, and shade of color represents strength of correlation). FT3, free triiodothyronine; FT4, free thyroxine; WBC, white blood cell; HGB, hemoglobin; PLT, platelet; Lys, lymphocytes; CRP, C-reactive protein; PCT, procalcitonin; ESR, erythrocyte sedimentation rate; PT, prothrombin time; APTT, activated partial thromboplastin time; FDP, fibrin degradation product; ALT, alanine aminotranspherase; AST, aspartate aminotransferase; Alb, albumin; TBil, total bilirubin; IBil, indirect bilirubin; Cr, serum creatinine; BUN, urea nitrogen. **(F)** *p < 0.05, **p < 0.01, ***p < 0.001.

Next, we analyzed the correlation of FT3 and clinical parameters to clarify the role of FT3 in COVID-19 progression. As illustrated, it can be seen that Lys, CRP, ESR, D-dimer, and albumin (ALB) significantly correlated with FT3 from T0 to T4. Among them, Lys and ALB positively correlated with FT3, whereas CRP, ESR, and D-dimer were negatively correlated with FT3. WBC at T4, hemoglobin (HGB) at T0 to T3, PLT at T0, and APTT at T3 were significantly positively correlated with FT3. PT at T0 to T1, FDP at T0 and T3, AST at T0–T1, BUN at T0–T1, and TBil at T1were significantly negatively associated with FT3 (**Figure 1**).

## Discussion

In the present study, we collected and evaluated the clinical data from 186 patients with COVID-19, which primarily included clinical characteristics and laboratory findings at multiple time points. The results of this study indicated that a lower FT3 level means a worse prognosis. Moreover, our data suggested that there may be a link between serum FT3 and some important markers of inflammation/coagulopathy/fibrinolysis, which is likely significant in the progression of COVID-19.

We compared the demographic characters, clinical symptoms, comorbidities, and outcomes of the ESS group and the non-ESS group. It likely proved that ESS may appear in the patients who were older and, together with hypertension or diabetes, especially the COVID-19 patients, were likely to have poorer outcomes. There were some common characters found with the previous studies that a greater proportion of patients were male, the most frequent symptoms were fever and cough, and the most common comorbidities were hypertension and diabetes (16–18).

In this study, the most important result was that a low FT3 level may suggest a poor prognosis, which has been reported in previous studies. The areas under the curve (AUCs) for predicting the severe disease was 0.809 for FT3 according to a study consisting of 149 COVID-19 patients (3). Another study in 191 patients with COVID-19 indicated a decreasing trend of FT3 with increasing COVID-19 severity (13). As stated similarly by a recent study, serum FT3 can predict COVID-19 mortality using the area under the receiver operating characteristic (ROC) curve, and ESS was associated with a 7.05 OR of mortality (15). Moreover, FT3 was thought to be an independent marker of the severity in patients with COVID-19 (14). Meanwhile, the non-survival patients had a lower level of serum TSH compared to the survival patients. A study consisting of 50 patients demonstrated that a more severe COVID-19 maybe linked with a lower TSH (19). Subsequently, the clinical data of 119 patients showed that TSH levels were significantly lower in patients with severe to critical disease than in those with non-severe disease (4). Moreover, what we know is that ESS is common in various acute and chronic conditions as a risk factor for the severity of diseases. However, it is notable that the pattern of FT3, FT4, and TSH changes in non-COVID-19 patients who suffer from ESS seems to be different from our study in COVID-19 patients. A study in non-COVID-19 patients with ESS showed that the acute physiology and chronic health evaluation II (APACHE II) score, an indicator of the severity of disease, was not significantly related to FT3 but significantly related to TSH (20). In trauma patients with multiple organ dysfunction syndrome (MODS), not only the level of FT3 but also the levels of total T3 and total T4 were significantly lower than that in non-MODS, and patients with low TSH have lower T3 scores and higher APACHE II scores (21). Therefore, the pattern of FT3 and TSH changes may be unique in the pathophysiological process of COVID-19.

Even though it has been assumed that low FT3 may have the predictive value, the mechanism of FT3 reduction is undefined. We speculate that the reasons are as follows. First, the inflammation response may take responsibility for the low FT3. Previous studies have shown that low FT3 is associated with systemic inflammation (13). It is suggested that COVID-19 could lead to severe inflammation. The results of the present study showed the relevant inflammation biomarkers with corresponding changes at early stages on admission (T0–T1), including the significant increase in WBC, CRP, PCT, and ESR, with the significant decrease in Lys, consistent with previous studies (3, 5, 13, 14, 22). Furthermore, we found that the changes were even more dramatic in the ESS group compared to the non-ESS group; the inflammatory responses seemed to be stronger. Moreover, our study indicated that CRP and ERS were negatively related to FT3, and Lys positively related to FT3 at all timepoints in the first 15 days after admission. Second, in terms of coagulation/fibrinolysis pathway, D-dimer, FDP, PT, and TBil inversely related to FT3 at one or more time points, and PLT and APTT were positively associated with FT3 at one or more time points. Meanwhile, there was a significant upward trend in the D-dimer, especially in COVID-19 with ESS (the minimum in period T2). A D-dimer level ≥2.0 μg/ml on admission was the optimum cutoff to predict in-hospital mortality for COVID-19 (23). Besides, a strong positive relationship between FT3 and ALB had been found, with a distinct decline in the plasma ALB in the COVID-19 patients (7, 24). ALB is a marker of liver function; the decreasing ALB means that liver function may have been impaired. The decrease in plasma ALB may also be related to increased excretion, meaning kidney function may also be involved (25), which was demonstrated by a negative relation between FT3 and BUN in the present study (24). ALB was not only a biomarker of liver function but also related to kidney injury. Levels of ALB may be affected by many factors and were significantly associated with the outcomes of COVID-19 (26, 27) and non-COVID-19 patients (28, 29).

What is surprising is that in the overall trend of laboratory findings of inflammation/coagulopathy/fibrinolysis in ESS patients at different time points, most parameters emerged a turning point at T3 (D9–D11 after admission), which is almost certainly a critical period related to prognosis. CRP, D-dimer, and fibrinogen (FIB) increased at the initial phase of SARS-COV-2 infection, with two peaks at day 5 and day 9 (30). CRP played an important role in inflammation, the tissue factor (TF) pathway, blood coagulation, endogenous fibrinolytic capacity, and the susceptibility of venous thromboembolism (31, 32). FIB was associated with inflammation, blood clotting, and fibrinolysis (33). Therefore, the levels of CRP and FIB could serve as the biomarkers of the pre-DIC phase. Many COVID-19 patients with thromboses and DIC have been reported, of whom the key features include mildly increased FIB and decreased PLT. Inflammatory responses to COVID-19 were thought to be one of the important causes of DIC, especially the cytokine storm. Thus, the association of FT3 and inflammation/coagulopathy/fibrinolysis suggest that low levels of FT3 may be another factor of DIC. We need deep analysis to demonstrate this association.

We are aware that our research may have three limitations. First, this study was a retrospective design, which provides a lower level of evidence than prospective and interventional studies. Second, our study only briefly describes the predictive value of low FT3 on the prognosis in patients with COVID-19, without in-depth exploration. Third, we did not analyze the DIC score and the direct association of FT3 with DIC. Further studies were needed to demonstrate our results.

In conclusion, COVID-19 patients with severe cases or who died have a lower FT3 level compared with non-severe or survival. Our research showed that FT3 was positively associated with Lys and ALB, and negatively related to CRP, ERS, and D-dimer. Despite some limitations, we can conclude that FT3 has a significant predictive value on the prognosis and is significantly related with inflammation/coagulopathy/fibrinolysis. Considerably more work needs to be done to illustrate details about the issue.

## Data Availability Statement

The datasets presented in this article are not readily available because the data used in this paper are from public health treatment center, which can only be obtained with the approval of relevant institutions. Requests to access the datasets should be directed to YZ, zhongyanjun@csu.edu.

## Ethics Statement

The study was approved by the institutional ethics board of the Second Xiangya Hospital of Central South University (No. 2020001). The ethics committee waived the requirement of written informed consent for participation. Written informed consent was not obtained from the individual(s) for the publication of any potentially identifiable images or data included in this article.

## Author Contributions

JD and YZ were involved in study design, interpreting data, statistical analysis, and writing of the manuscript. SZ, FP, QZ, and YL were involved in collecting data. All authors contributed to the article and approved the submitted version.

## Conflict of Interest

The authors declare that the research was conducted in the absence of any commercial or financial relationships that could be construed as a potential conflict of interest.

## Publisher’s Note

All claims expressed in this article are solely those of the authors and do not necessarily represent those of their affiliated organizations, or those of the publisher, the editors and the reviewers. Any product that may be evaluated in this article, or claim that may be made by its manufacturer, is not guaranteed or endorsed by the publisher.

## Abbreviations

COVID-19, coronavirus disease 2019; SARS-CoV-2, severe acute respiratory syndrome coronavirus 2; DIC, diffuse intravascular coagulation; ESS, euthyroid sick syndrome; ACE2, angiotensin-converting enzyme 2; APACHE II, acute physiology and chronic health assessment II; FT3, free triiodothyronine; FT4, free thyroxine,T3, triiodothyronine; T4, thyroxine; TSH, thyroid-stimulating hormone; PaO2, partial pressure of arterial oxygen; FiO_2_, fraction of inspired oxygen; WBC, white blood cell; HGB, hemoglobin; PLT, platelet; Lys, lymphocytes; CRP, C-reactive protein; PCT, procalcitonin; ESR, erythrocyte sedimentation rate; PT, prothrombin time; APTT, activated partial thromboplastin time; FDP, fibrin degradation product; FIB, fibrinogen; ALT, alanine aminotranspherase; AST, aspartate aminotransferase; ALB, albumin; TBil, total bilirubin; IBil, indirect bilirubin; Cr, serum creatinine; BUN, urea nitrogen; MODS, multiple organ dysfunction syndrome; TF, tissue factor.
